# Supplementary figures and images for: Identification of hub genes and small-molecule compounds in medulloblastoma by integrated bioinformatic analyses
Source: PeerJ. 2020 Apr 14;8:e8670. doi: 10.7717/peerj.8670 (PMC7164431; doi:10.7717/peerj.8670)

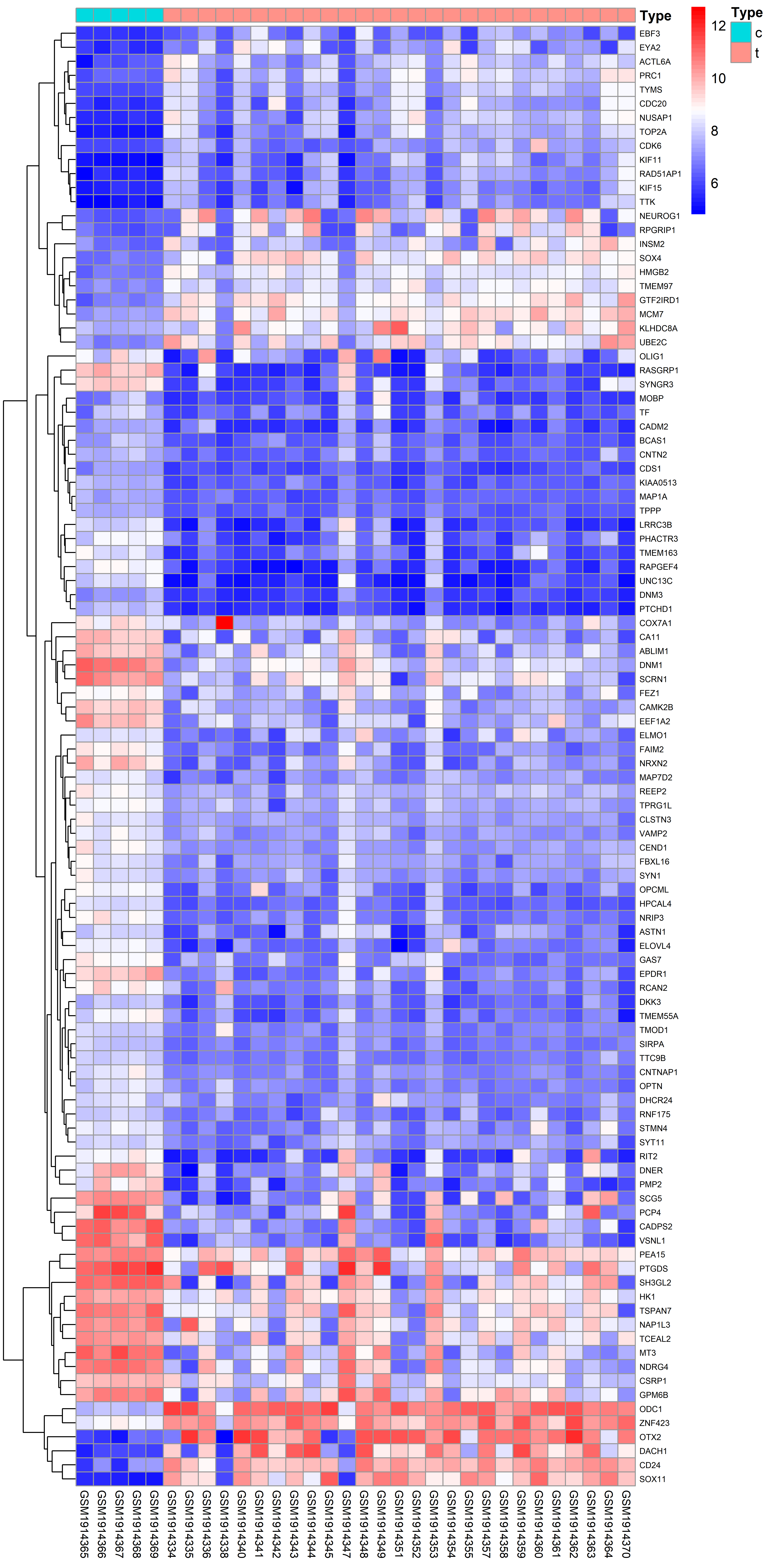

Supplement: Supplemental Information 1 — Red: upregulated; Green: downregulated [file peerj-08-8670-s001.png]
